# Dermal fibroblasts have different extracellular matrix profiles induced by TGF-β, PDGF and IL-6 in a model for skin fibrosis

**DOI:** 10.1038/s41598-020-74179-6

**Published:** 2020-10-14

**Authors:** Pernille Juhl, Sandie Bondesen, Clare Louise Hawkins, Morten Asser Karsdal, Anne-Christine Bay-Jensen, Michael Jonathan Davies, Anne Sofie Siebuhr

**Affiliations:** 1 Department of Biomedical Sciences, University of Copenhagen, Copenhagen, Denmark; 2 Biomarkers and Research, Nordic Bioscience, Herlev hovedgade 207, 2730 Herlev, Denmark

**Keywords:** Cell biology, Rheumatic diseases

## Abstract

Different stimulants might induce different extracellular matrix profiles. It is essential to gain an understanding and quantification of these changes to allow for focused anti-fibrotic drug development. This study investigated the expression of extracellular matrix by dermal fibroblast mimicking fibrotic skin diseases as SSc using clinically validated biomarkers. Primary healthy human dermal fibroblasts were grown in media containing FICOLL. The cells were stimulated with PDGF-AB, TGF-β1, or IL-6. Anti-fibrotic compounds (iALK-5, Nintedanib) were added together with growth factors. Biomarkers of collagen formation and degradation together with fibronectin were evaluated by ELISAs in the collected supernatant. Immunohistochemical staining was performed to visualize fibroblasts and proteins, while selected gene expression levels were examined through qPCR. TGF-β and PDGF, and to a lesser extent IL-6, increased the metabolic activity of the fibroblasts. TGF-β primarily increased type I collagen and fibronectin protein and gene expression together with αSMA. PDGF stimulation resulted in increased type III and VI collagen formation and gene expression. IL-6 decreased fibronectin levels. iALK5 could inhibit TGF-β induced fibrosis while nintedanib could halt fibrosis induced by TGF-β or PDGF. Tocilizumab could not inhibit fibrosis induced in this model. The extent and nature of fibrosis are dependent on the stimulant. The model has potential as a pre-clinical model as the fibroblasts fibrotic phenotype could be reversed by an ALK5 inhibitor and Nintedanib.

## Introduction

Skin fibrosis can cause serious problems and changes the organization of the skin and is the result of e.g. deregulated wound healing or an underlying disease. There are several skin fibrotic disorders as systemic sclerosis (SSc), nephogenic systemic fibrosis, eosinophilic fasciitis, scleromyxedema, and more. The example and focus in this manuscript is on SSc.

SSc is a complicated disease with its pathogenesis including a combination of excessive fibrosis, vascular damage and immune dysregulation^1^. This is further complicated for SSc as the etiology is still unknown and SSc is considered incurable^2^. Generally, potential drugs can be divided into four categories based on their target; fibrotic, inflammatory, immunity and vascular^3^. SSc patients are generally diagnosed based on the extent of skin thickening, but the disease progression can affect different organs in individual patients, primarily by fibrosis^1^. Fibrosis is caused by chronically activated myofibroblasts producing excessive amounts of extracellular matrix (ECM), especially collagens and fibronectin^4^. Especially interstitial matrix collagens as type I, III, and VI collagens are known to be upregulated during fibrosis. The primary function of the fibroblasts is maintaining tissue homeostasis through regulation of the ECM^5^. However, continuous fibroblast activation is facilitated by cytokines and growth factors such as interleukin 6 (IL-6), platelet-derived growth factor (PDGF), and transforming growth factor-beta (TGF-β), which are released by the immune and vascular systems. This results in excessive ECM production, especially different collagens, manifested as skin and internal organ fibrosis.

Several growth factors are upregulated in skin fibrosis and activates fibroblasts. TGF-β is perhaps the most studied growth factor in fibrosis. It has been shown to induce differentiation to myofibroblasts and to stimulate ECM production directly^3,6^. Activated immune cells, such as macrophages, together with fibroblasts are the main sources of TGF-β production, with these cell types known to be upregulated in SSc^2^. Immune cells are also known to secrete cytokines, such as interleukins (for example IL-6), which can also stimulate fibroblasts^2^. IL-6 have been implicated in skin fibrosis and is known to correlate with skin thickness^7,8^. Vascular damage induces the release of growth factors, such as PDGF. PDGF has been shown to be upregulated in SSc and is responsible for inducing fibroblast proliferation^9^.

Taking drugs from research to clinical trials is a difficult, expensive and time-consuming affair, and there is a high unmet need to predict the clinical response of potential treatments based on pre-clinical outcomes^10^. Primary dermal fibroblasts have been widely used to screen treatments to confirm the mode of action^11,12^. However, fibroblasts change phenotype, when they are plated on plastic surfaces, such as tissue culture plates^13^. This phenotype change has traditionally been overcome by using 3D cultures^14^. Recently, macromolecular crowding has been examined as a means to imitate the crowded in vivo environment surrounding the fibroblasts^15–17^. Macromolecular crowding involves the use of a soluble macromolecule to occupy space and thereby exclude volume^18^. This affects the concentration and time course of the appearance of soluble components such as proteins and proteases^15^.

As the fibroblast is the key effector cell of fibrosis, halting their chronic activation has been one of the main targets for drug development in skin fibrosis diseases as SSc. As TGF-β is one of the most studied growth factors related to fibroblast activation, it has also been the focus of treatments trying to prevent fibroblast activation^19^. Current drugs used or tested in SSc have often previously been approved for other fibrotic or rheumatic diseases and target fibrotic or inflammatory pathways. Treatments approved for interstitial pulmonary fibrosis, which targets TGF-β and PDGF are now tested in SSc. Fresolimumab, a humanized anti-TGF-β antibody, has shown positive results regarding SSc^20^, while nintedanib, a tyrosine kinase inhibitor (blocking PDGF), has been tested and approved for SSc with interstitial lung disease (ILD)^21^. Tocilizumab, an anti-IL-6 receptor antibody, which is approved for rheumatoid arthritis, is also being tested and has been shown to change the phenotype of the fibroblasts of the treated patients^22^. Both treatments have shown a reduction in the lung, but not skin, fibrosis in clinical trials, even though an improvement of lung function was only the primary outcome for nintedanib^23^.

Clinical trial design, especially within SSc, has made significant progress and together with precision medicine tools, have renewed hope for SSc therapeutics^3^. Precision medicine tools, such as translational biomarkers, together with better pre-clinical models, could add further value to the field. There is still an urgent need to develop pre-clinical models applicable for the skin fibrosis in SSc further and to pair them with translational tools. However, to achieve this need and better understand the development of fibrotic diseases, particularly SSc, greater mechanistic information is required concerning the fibroblasts’ response to stimulation. This is also important to test the therapeutic potential of pharmaceutical compounds on collagen production and determination of translational biomarkers.

In this study, we examined a model of fibrosis to understand the mode of action for single cytokine stimulation and co-stimulation of cytokines as well as to examine its potential to investigate anti-fibrotic treatments for skin fibrosis. PDGF and IL-6 were the targets of two recent clinical trials in SSc (nintedanib and tocilizumab, respectively) while TGF-β is the main growth factor in activating fibroblasts. The mode-of-action of these cytokines were examined. Their ability to activate fibroblasts and create an ECM mimicking skin fibrosis was investigated. We further investigated iALK5 and nintedanib as a therapeutic intervention to reverse the development of a fibrotic phenotype. Gene expression was used to understand the genes affected by the stimulation together with biochemical markers to examine protein levels. Assessing the alteration of gene expression together with biochemical markers could be useful in both pre-clinical and clinical models to understand and monitor the effect of treatments.

The objective of this study was to examine Ficoll-crowded human dermal fibroblasts as a model to test the response of these cells to fibrotic (TGF-β), inflammation (IL-6) and vascular stimulation (PDGF) alone, and in combination with known inhibitors.

## Results

### Single cytokine stimulation

**Figure 1:**
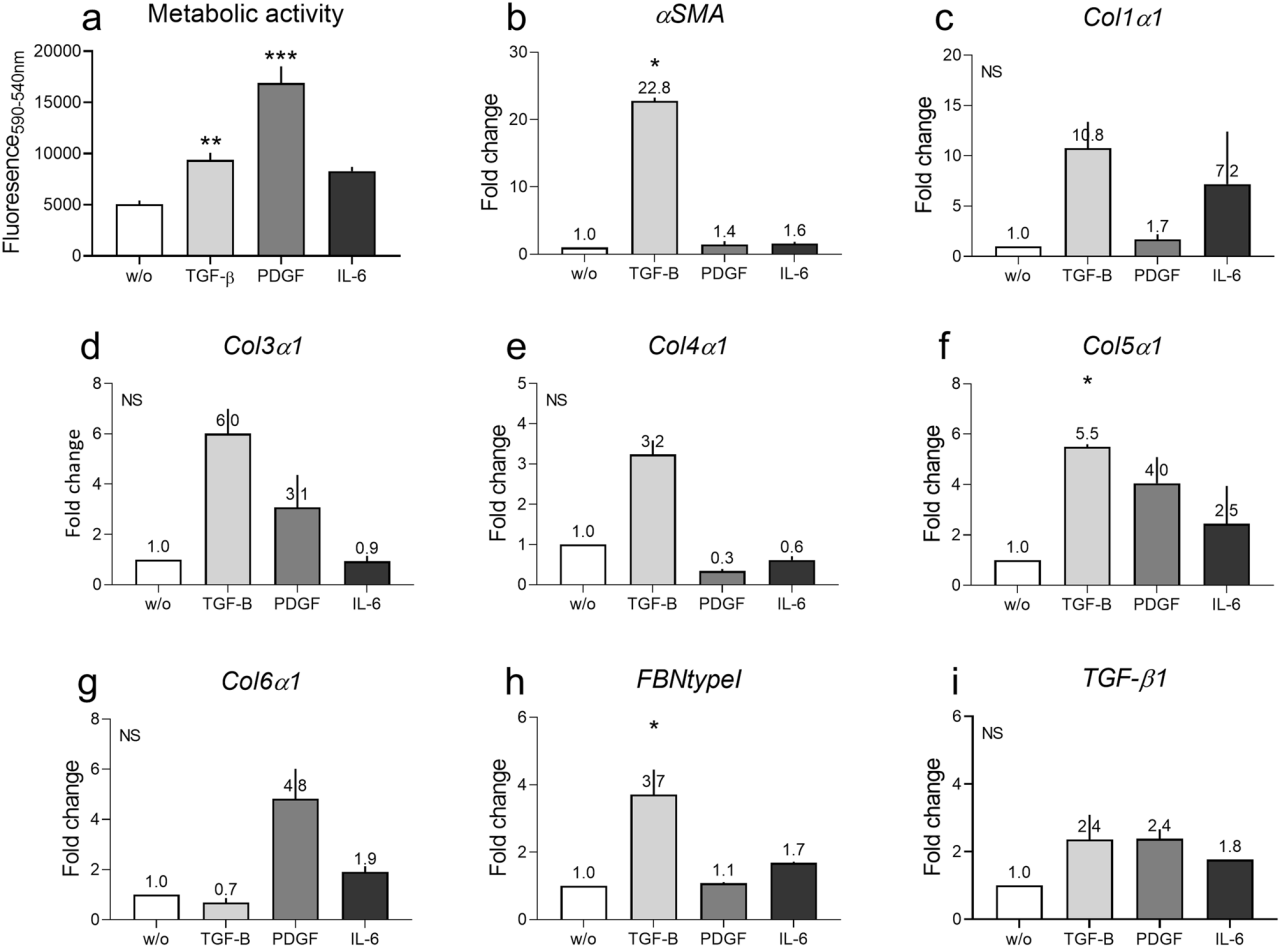
Single cytokine stimulation can activate dermal fibroblasts. The effect of TGF-β, PDGF or IL-6 (with sIL-6R) on the metabolic activity and mRNA levels of selected genes in healthy dermal fibroblasts. (**a**) Metabolic activity of fibroblasts after 14 days of stimulation. (**b**–**i**) Gene expression 3 days of stimulation. (**b**) αSMA. (**c**) Type I collagen (Col1α1). (**d**) Type III collagen (Col3α1). (**e**) Type IV collagen (Col4α1). (**f**) Type V collagen (Col5α1). (**g**) Type VI collagen (Col6α1). (**h**) Fibronectin type I (FBNtypeI). (**i**) TGF-β1. Four technical replicates were used to assess the metabolic activity and two technical replicates were used to assess gene expression. Data are shown as mean ± SD. Data were analyzed by Kruskal–Wallis test^53^. *P < 0.05, **P < 0.01, ***P < 0.001.

Initial studies examined the effects of single cytokine stimulation on healthy human dermal fibroblasts in a crowded environment. Phenotypical cytokines of Fibrosis (TGF-β), vascular (PDGF) and inflammation (IL-6) were used to activate fibroblasts. Firstly, their effect on intracellular mechanisms was examined. An increase in the metabolic activity of the fibroblasts was observed in each case, which was significant in experiments with TGF-β (P = 0.003) and PDGF (PDGF: P < 0.0001) (Fig. 1A). All three cytokines increased the mRNA levels of several genes after 3 days of stimulation: TGF-β increased αSMA (P = 0.04), type I, III, IV and V collagen, fibronectin (P = 0.04) and TGF-β1 genes while downregulating type VI collagen (Fig. 1B–I). PDGF increased type III, V and VI collagen and TGF-β1 genes expression, and downregulated type IV collagen. IL-6 increased type I, V and VI collagen and TGF-β1 gene expressions.

**Figure 2:**
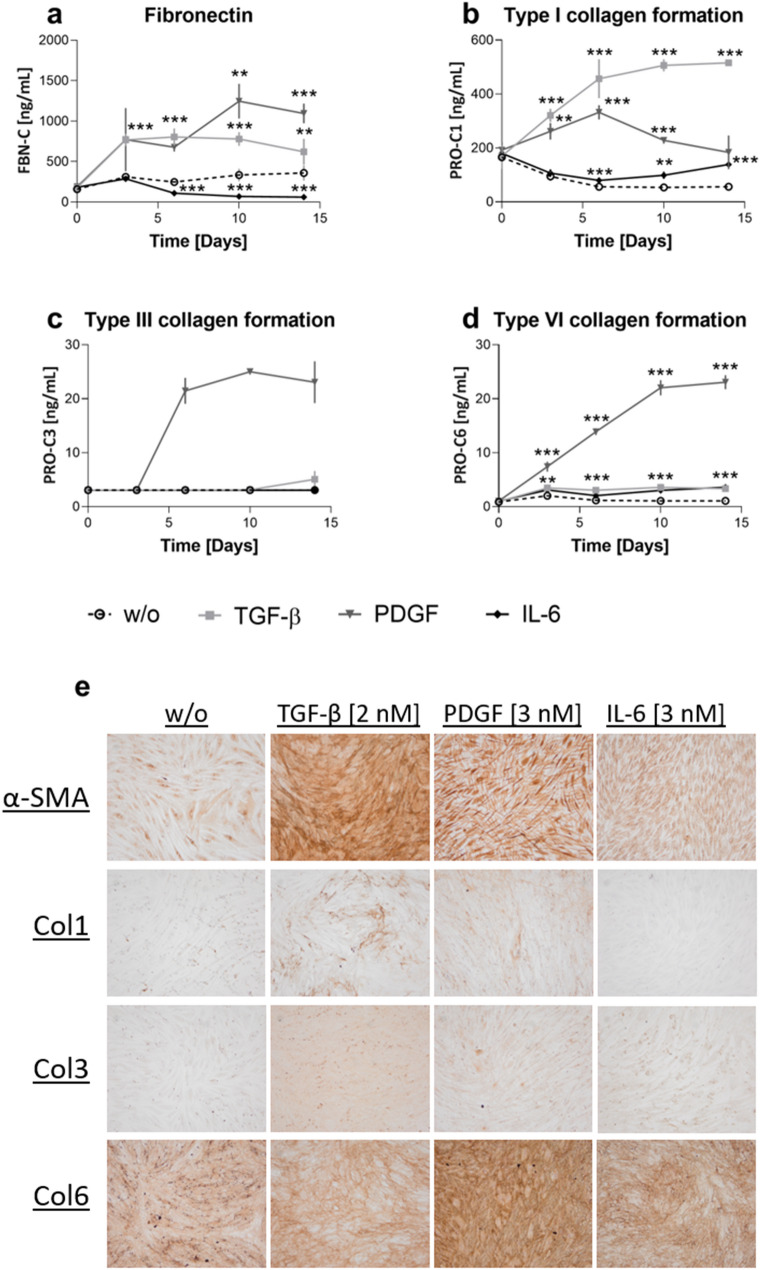
Single cytokine stimulation can stimulate protein production. The effect of TGF-β, PDGF or IL-6 (with sIL-6R) on protein production in healthy dermal fibroblasts. (**a**–**d**) Protein levels measured in the supernatant. (**a**) Fibronectin levels over time. (**b**) Type I collagen formation over time. (**c**) Type III collagen formation over time. (**d**) Type IV collagen formation over time. (**e**) Staining of αSMA and type I, III, and VI collagen at the bottom of the well. Four technical replicates were used to assess protein levels over time. Data are shown as mean ± SD. Data were analyzed by two-way ANOVA with Dunnett’s multiple comparisons test^54^. *P < 0.05, **P < 0.01, ***P < 0.001.

The protein levels of fibronectin and type I, III, IV, V and VI collagen excreted into the cell medium were investigated in response to the cytokines mentioned above. TGF-β increased fibronectin and type I and VI collagen formation from day 3 (FBN-C, PRO-C1 and PRO-C6: P ≤ 0.0001, Fig. 2A–D). PDGF increased type I and VI collagen formation from day 3, and fibronectin and type III collagen formation from day 6 (PRO-C1: P = 0.001, PRO-C6: P = 0.003, FBN-C: P < 0.0001). Type III collagen formation could not be statistically analyzed as the control (w/o) samples were below the lower limit of quantification, thus eliminating any variation. However, a 6.9- to 8.2-fold change was observed for PDGF stimulation compared to the control (w/o). In addition, PDGF increased cross-linked type III collagen formation up to 21-fold (Supplementary Information). IL-6 increased type I and VI collagen formation from day 6 and 3, respectively (PRO-C1: P = 0.0009, PRO-C6: P = 0.009) and decreased fibronectin from day 6 (FBN-C: P < 0.0001).

Measurements of type IV and type V collagen formation and the degradation biomarkers did not provide a characteristic change or fingerprint for the specific cytokine stimulation. Type IV collagen formation was not affected by cytokine stimulation, as the expression of this protein was comparable to that seen in untreated fibroblasts. Type V collagen formation and the degradation biomarkers were below the lower level of quantification.

Immunohistochemistry showed that TGF-β, PDGF and IL-6 stimulation increased αSMA in the fibroblasts (Fig. 2E). It further showed that the fibroblast organization was dependent on the nature of the stimulation, as fibroblasts appeared multi-layered when stimulated with TGF-β and PDGF. The wells were further stained for type I, III and VI collagens to verify that the collagens produced were used to create an ECM rich environment laid down on the bottom of the well. Type I collagen was excreted and deposited in the well in response to TGF-β and PDGF stimulation. Type III and VI collagen were produced and adhered to the well in response to all treatments.

#### Effects of therapeutic compounds on ECM synthesis and turnover

To examine the model’s capacity to act as a pre-clinical screen for therapeutic compounds, an ALK5 inhibitor (iALK5) and nintedanib were tested. For both inhibitors, their capacity to inhibit further collagen formation was investigated by first activating the fibroblasts for seven days and then adding the inhibitor. Their ability to block total collagen production was also tested by treating the fibroblasts with the compounds from day 0 (Supplementary Information).

**Figure 3:**
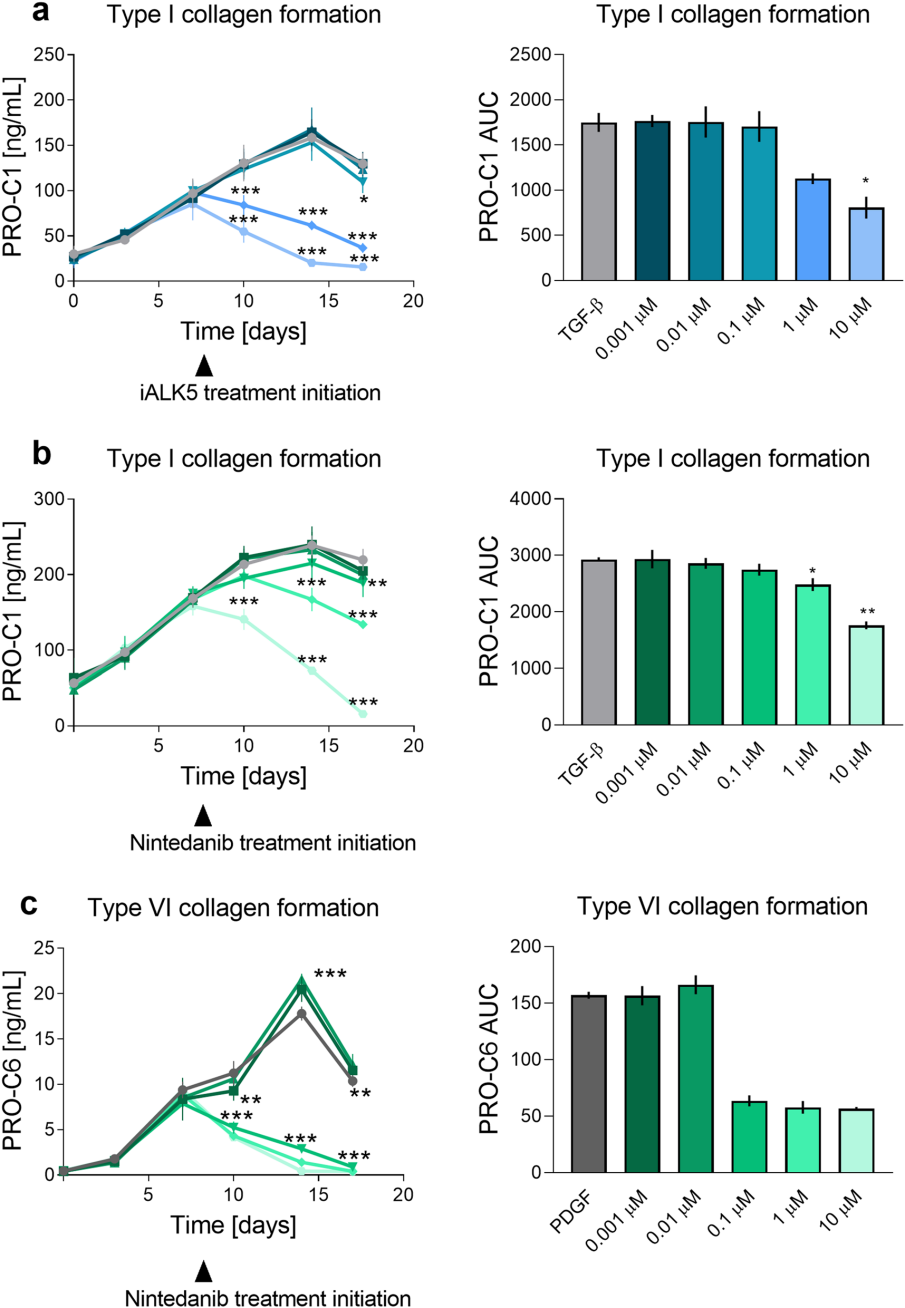
Fibrosis can be modulated by currently-used therapeutic drugs. Pre-stimulated (7 days) cells received an anti-fibrotic compound together with either TGF-β or PDGF. Both the collagen levels over time (left column) and the AUC (right column) are depicted. (**a**) Type I collagen formation in response to TGF-β and iALK5 treatment form day 7. (**b**) Type I collagen formation in response to TGF-β and nintedanib treatment form day 7. (**c**) Type VI collagen formation in response to PDGF and nintedanib treatment form day 7. Four technical replicates were used to assess protein levels over time. Tecknical replicates are 4 and data are shown as mean ± SD. Data were analyzed by two-way ANOVA with Dunnett’s multiple comparisons test^54^ and AUC^52^. *P < 0.05, **P < 0.01, ***P < 0.001.

The addition of iALK after 7-day TGF-β stimulation showed only a non-significant, dose-dependent, decrease in metabolic activity (Supplementary Information). iALK5 (1 µM) also showed a tendency to decrease the gene expression of αSMA, type I, III, IV and V collagen, fibronectin and TGF-β, while iALK5 increased type VI collagen mRNA levels (Supplementary Information). A decrease in type I collagen formation was observed with 1 and 10 µM iALK5 from day 10 (P < 0.0001, Fig. 3A). The addition of iALK5 (1 and 10 µM) together with TGF-β from day 0, decreased type I collagen formation from day 3 to the level of the vehicle and 0.1 µM iALK5 decreased the levels from day 7 (0.1–10 µM: P ≤ 0.0001, Supplementary Information).

Nintedanib was added together with either TGF-β or PDGF. Addition of nintedanib after 7-day TGF-β pre-treatment showed that nintedanib dose-dependently decreased metabolic activity. The highest dose (10 µM) eliminated any metabolic activity (1 µM: P = 0.03 10 µM: P = 0.002, Supplementary Information). Addition of 1 µM nintedanib showed a tendency to decrease type III, IV and V collagen, fibronectin and TGF-β genes, while it increased type VI collagen, and did not alter αSMA and type I collagen gene expression (Supplementary Information). Nintedanib (1 µM and 10 µM) reduced type I collagen protein formation significantly, while 0.1 µM had a lesser effect on type I collagen levels (day 14; 0.1 µM: P = 0.04, 1–10 µM: P < 0.0001, Fig. 3B). Stimulation with TGF-β in the presence of nintedanib from day 0, showed that nintedanib significantly decreased type I collagen formation in a dose-dependent manner (day 3; 0.1 µM: P = 0.003, 1–10 µM: P < 0.0001, Supplementary Information). Nintedanib (1 µM) decreased type I collagen production by about 50%, while 10 µM reduced the levels to those of the vehicle. Nintedanib (0.001–0.1 µM) also reduced type I collagen formation (day 3; 0.1 µM: P = 0.001, 1–10 µM: P < 0.0001).

Addition of nintedanib (0.1–10 µM) after 7-day PDGF pre-treatment decreased metabolic activity (10 µM: P = 0.04) and type VI collagen formation (day 14; 0.1–10 µM, P < 0.0001, Fig. 3C). Stimulating with PDGF in the presence of nintedanib (0.1–10 µM) from day 0 decreased type VI collagen formation to the level of the vehicle (day 7; 0.1–10 µM: P < 0.0001 Supplementary Information).

## Discussion

There is a great need for pre-clinical models which imitate disease pathogenesis as well as predict the effect of potential drugs. Fibroblasts are the primary effector cell in fibrosis and dermal fibroblasts are responsible for the excessive skin fibrosis found in diseases as SSc. However, their change in behavior when cultured on plastic surfaces complicates in vitro studies^13^. Several reports have proposed stimulating primary fibroblasts in a crowded macromolecular environment to help overcome some of these difficulties^15–18,24^. In this model, we combined the macromolecular crowding with dermal fibroblasts and translational biomarkers. It has previously been reported that biomarkers of type I, III and VI collagen formation are elevated in SSc patients^25–28^. In the current study, we show that this pre-clinical model may be used for screening potential drugs. Nintedanib could reverse the fibrotic phenotype induced by TGF-β and/or PDGF, and iALK5 could reverse TGF-β induced fibrosis. Furthermore, the data show that TGF-β, PDGF and IL-6 induced different fibrotic phenotypes of fibroblasts generating distinct ECM profiles.

We found that the three cytokines tested, stimulated fibroblasts differently: TGF-β, PDGF and IL-6 were found to produce different ECM profiles, both at the gene and protein levels. We, therefore, argue that fibrosis is not just fibrosis. Different stimulants result in alternative matrix compositions, and an in-depth analysis is necessary to understand the effects of stimulation on fibrosis. TGF-β is known to be a key growth factor for fibrogenesis and is a commonly used cytokine to stimulate fibroblasts to a fibrotic state. It has often been reported to be upregulated in skin fibrosis, such as SSc^29,30^. We observed an increase in both type I collagen and fibronectin gene and protein levels. An increase of αSMA, type III, IV and V collagen and TGF-β genes were further observed. Even though there was an increase in type III collagen genes, we did not observe an increase in protein levels. This may be due to differential processing. A marked increase in type I collagen has been reported in several studies^12,31^.

PDGF was found to increase metabolic activity to the greatest extent. PDGF is known to increase fibroblast proliferation, and an increase in metabolic activity could reflect increased numbers of fibroblasts. PDGF is known to induce fibroblast differentiation into myofibroblasts as well as stimulating collagen production in fibrotic diseases such as SSc^32^. In our model, PDGF increased both type III and VI collagen gene and protein expression. It also increased type V collagen and TGF-β genes and fibronectin protein levels. This validates PDGF’s role as a growth factor that drives ECM production. Several cells produce PDGF, including platelets^9^. They are known to be activated in SSc, especially in response to vascular damage where they produce both PDGF and TGF-β^33^. It can, therefore, be hypothesized that the collagen synthesis can be a direct response to vascular damage through PDGF and that this gives a specific phenotype of a type VI collagen-rich ECM.

TGF-β and PDGF have previously been shown to produce an equal amount of type I collagen^31^. In our study, TGF-β appeared to be the most potent cytokine to activate several genes, but PDGF stimulated an increase in the formation of type III and VI collagen protein formation, distinct from the TGF-β profile. The collagens showed to be dispositioned in the wells. These results are supported by the increased collagen formation biomarkers, while degradation biomarkers were not changed, giving a net increase in overall collagen.

IL-6 is known to be increased in SSc, and it is involved in the pathogenesis of this disease^8,34,35^. IL-6 was able to increase type I collagen gene and protein levels while it did not alter fibronectin genes and decreased protein levels. This question’s the role of IL-6 in modulating the activity of healthy fibroblasts. Several studies have shown IL-6 to be pro-fibrotic and be a potential target for treatment, with tocilizumab being examined as a treatment for SSc^22,23,36^. Studies on hepatocytes have been reported to result in reduced extracellular fibronectin levels^37^. Does IL-6 have an anti-fibrotic effect and if so, how does it switch to be a pro-fibrotic agent and a target of anti-fibrotic treatments? More experiments are needed to understand the different roles of cytokines in healthy and diseased cells. Tocilizumab has been shown to change the phenotype of SSc dermal fibroblasts but not healthy cells^22^, indicating that IL-6 plays an important role in SSc fibroblasts. A combination of IL-6 and the IL-6 receptor have previously shown to indirectly increase fibrosis through Gremlin protein in dermal fibroblasts from SSc patients^38^. Indicating that IL-6 alone are perhaps not fibrotic, but it rather elevates other autocrine fibrotic proteins.

Anti-fibrotic compounds such as iALK5, a TGF-β receptor inhibitor, and nintedanib were able to reduce the amount of collagen produced, consistent with previous findings^39^. iALK5 was able to reduce TGF-β type I collagen formation, and nintedanib was able to reduce type I and VI collagens formation induced by TGF-β and PDGF, respectively. Nintedanib has been tested in pre-clinical models of both skin fibrosis in SSc^11^ and interstitial pulmonary fibrosis (for which it was initially approved)^40^ with successful results and in a clinical study of SSc where it showed an improvement of lung function but not skin fibrosis (mRSS)^21^. The same assessments were not used in pre-clinical and clinical trials. We, therefore, argue that pre-clinical models and clinical trials should include the same assessments if possible. Our model has the potential to be a pre-clinical model to examine anti-fibrotic compounds for dermal fibrosis and SSc with the translational biomarkers described.

Healthy and SSc dermal fibroblasts are known to behave differently, making it hard to predict the modes of action of drugs using healthy dermal fibroblasts. However, fibroblasts from SSc patients represent a limited source of materials as they are isolated from a small sample population.

The combination of a pre-clinical model and translational biomarkers is a powerful tool to follow potential anti-fibrotic drugs. The model’s imitation of SSc opens the field for the screening of potential anti-fibrotic drugs in a more relevant and hopefully accurate model. Here, we showcase a pre-clinical model with evidence of collagen cross-linking, deposition of ECM and an understanding of the modes of action of cytokines, together with quantifiable translational biomarkers. However, the processes that convert healthy dermal fibroblasts into SSc fibroblasts are still not fully understood.

This study has limitations. As we did not have access to diseased skin fibroblasts as from SSc patients, it was not possible to assess the similarities to the healthy fibroblasts. Further, the gene and protein expression did not always align. There appear to be differences in the timing of transcription of mRNA and the cleavage of pro-peptides, and we have yet to map these procedures to understand these differences. Furthermore, the mRNA levels were adjusted for cell numbers through overall RNA levels. The same was not possible for measurements of conditioned media. As PDGF cells are known to proliferate more, the increased protein levels might be due to more cells being present and not increased synthesis by single cells.

In conclusion, healthy dermal fibroblasts in a crowded environment together with translational biomarkers could be an essential pre-clinical model of skin fibrosis for use in diseases as SSc. In this model, it is possible to assess the fibroblasts in a crowded environment and how different pathways lead to altered patterns of fibrosis. Cytokines characteristic of fibrosis (TGF-β), vascular damage (PDGF) and immune activation (IL-6) induce very different ECM compositions and drugs targeting specific pathways should, therefore, examine the modulation of specific ECM materials, and not just total ECM levels. Co-stimulation by cytokines may be a way to create more SSc-like fibroblasts. The model described here does, however, have potential as a pre-clinical model, and may be used to test novel anti-fibrotic compounds.

## Methods

### Cell culture

Healthy human primary dermal fibroblasts (Adult CC-2511, Lonza/Cell applications; N = 2) were cultured at low passage (N = 6–9). Both donors were females with one donor being of Persian descent (28 years old) and the other was Caucasian (37 years old). The two patients involved in this study gave informed consent. Experimental protocols used were in accordance with GRP regulations and guidelines. Protocols were approved by Nordic Bioscience A/S ethics committee.

The cells were seeded in a 48-well plate at 30,000 cells per well in Dulbecco’s modified Eagle’s medium (DMEM) + Glutamax (Gibco) with 1% penicillin–streptomycin (P/S) and 10% fetal calf serum (FCS) 37 °C, 5% CO_2_. After 1 day, the cells were serum-starved for an additional day with media containing 0.4% FCS, before starting the treatment. IL-6 [3 nM] (+/− sIL-6R [0.03 nM]), PDGF-AB [3 nM] and TGF-β1 [2 nM] were examined separately. Anti-fibrotic compounds were tested in response to vascular or fibrotic stimulation. ALK-5 (TGF-β receptor) inhibitor (iALK-5 [0.001–10 µM]) was added together with TGF-β [1 nM] with or without seven days pre-treatment with TGF-β. Nintedanib [0.001–10 µM] was added together with either 1 nM TGF-β or 3 nM PDGF with or without seven days pre-treatment with TGF-β or PDGF, respectively. Non-stimulated cells were used as a control (w/o). Media contained Ficoll (Ficoll 70 [56 mg/mL] + 400 [38 mg/mL]) and l-ascorbic acid 2-phosphate (1.5%). The medium was exchanged twice a week with treatment added each time. No rinse was performed between the medium changes. Supernatants were collected for biomarker measurement and kept at − 20 °C until analysis. The metabolic activity was assessed at the beginning and end of the experiments using the AlamarBlue assay. The experiments were terminated on day 14 (cytokine stimulation) or 17 (effect of treatment).

### Cell viability

The effect of stimulants and treatments on cell metabolic activity was examined by the AlamarBlue assay, following the manufacture’s guidelines. Cells were incubated with media containing 10% AlamarBlue for two h at 37 °C, 5% CO_2_. The conditioned media were transferred to a black 96-well plate with the fluorescence read using 540 nm as excitation wavelength and 590 nm as emission wavelength.

### Enzyme-linked immunosorbent assays

Biomarkers of ECM turnover levels were measured at every media change throughout the experiments using validated competitive ELISAs (Nordic Bioscience). Type I, III, IV, V and VI collagen formation (PRO-C1^41^, PRO-C3^42^, PC3X, PRO-C4^43^, PRO-C5^44^, PRO-C6^45^) and degradation (C1M^46^, C3M^47^, C4M^48^, C5M^49^, C6M^50^) biomarkers together with a biomarker of fibronectin (FBN-C^51^) were measured. The ELISAs were run accordingly to the manufacturer’s protocol. A standard curve was generated for each assay through a four-parametric model from which the sample concentrations were calculated. Values below the detection limit were assigned the lower limit of detection.

### Immunohistochemistry analysis

Cells were seeded on 4-well Millicell EZ slides (Millipore) and stimulated as described above. At the end of stimulations, cells were fixed using 4% formaldehyde for two h.

The slides were washed three times in PBS, then 600 µl peroxidase-block was added and incubated for 10 min, followed by a wash in PBS. Slides were further blocked in 2% skim milk for 15 min. Mouse anti-type I, III or VI collagen or anti-α-SMA (diluted 1:500) was added to the slides and incubated at 4 °C for 20 h. After three washes, Dako HRP-labelled X anti-mouse (1:5000) was added, and the slides incubated for 30 min before being washed three times. The substrate containing chromogen (1 drop chromogen to 1 ml substrate) was added to the slides and incubated for 1–15 min. Slides were washed three times in PBS and counterstained in Mayer’s hematoxylin for 12 s, which was then removed by rinsing with tap water and left to dry. Pictures were taken with an Olympus DP71 digital camera connected to an Olympus BX60 microscope.

### qPCR

Cells were lysed with the RNeasy Lysis Buffer (RLT buffer) 3 days after stimulation. RNA was purified using a RNeasy Mini Kit (Qiagen) according to the manufacturer’s instructions. cDNA was generated by using the sensiFAST cDNA synthesis Kit (Bioline) according to the manufacturer’s instructions. The RNA quality was assessed by the absorbance ratio of 260/280 nm while the quantity was assessed by examining the absorbance ratio of 260/230 nm and normalized to 250 ng of total RNA per reaction using RNase free water^11^. The expression of mRNA was assessed by qPCR using the primer sequences outlined in Table 1. Samples were prepared using the sensiFAST SYBR Hi-ROX Kit (Bioline).

**Table 1:** Overview of primers for qPCR.

Gene	Forward	Reverse
α Smooth muscle actin	GCTGTTTTCCCATCCATTGTG	CCTCTTTTGCTCTGTGCTTC
Collagen1a1	CTGTAAACTCCCTCCATCCC	GTCCATGTGAAATTGTCTCCC
Collagen3α1	CTGGGGAATGGAGCAAAAC	AAAGCAAACAGGGCCAAC
Collagen4α1	ACGACATCATCAAAGGGGAG	ACCCACCAATCCTGTAACAC
Collagen5α1	ACCACCAAATTCCTCGACC	CCTCAAACACCTCCTCATCC
Collagen6α1	ATCGGACCTAAAGGCTACC	TTCTCCCCTTTCACCCATC
Fibronectin	GGACCAGGACCAACAAAAAC	AGACACTAACCACATACTCCAC
TGF-β	GGAAATTGAGGGCTTTCGCC	CCGGTAGTGAACCCGTTGAT
18S	GTAACCCGTTGAACCCCATT	CCATCCAATCGGTAGTAGCG

Relative mRNA concentrations of the genes of interest were normalized to the relative mRNA of the housekeeping gene 18S. Differences were calculated with the comparative Ct method for each target gene with the results expressed as a fold increase over the control^11^.

### Statistics

The biomarker levels are displayed as mean ± SD. Four technical replicates are used to assess the metabolic activity, and two technical replicates are used to assess gene expression. The area under the curve was calculated based on the biomarker measurement of each technical replicate.

P-values ≤ 0.05 were considered statistically significant. Graphical illustrations, Area Under the Curve (AUC)^52^, Kruskal Wallis test^53^ and two-way ANOVA with Dunnett’s multiple comparisons test^54^ were performed using GraphPad Prism version 8.

## Supplementary information

Supplementary Information.

## Data Availability

The datasets generated during and/or analyzed during the current study are available from the corresponding author on reasonable request.
