# Supplementary material for: Dermal fibroblasts have different extracellular matrix profiles induced by TGF-β, PDGF and IL-6 in a model for skin fibrosis
Source: Sci Rep. 2020 Oct 14;10:17300. doi: 10.1038/s41598-020-74179-6 (PMC7560847; doi:10.1038/s41598-020-74179-6)
Supplement: Supplementary file 1 — Supplementary Information. [file 41598_2020_74179_MOESM1_ESM.pdf]

**SUPPLEMENTARY MATERIAL**

Title: Dermal fibroblasts have different extracellular matrix profiles induced by TGF-β, PDGF and IL-6 in a model for skin fibrosis

Pernille Juhl, MSc[1,2*], Sandie Bondesen, MSc [1, 2], Clare Louise Hawkins, Professor, PhD[1], Morten Asser Karsdal, PhD[2], Anne-Christine Bay-Jensen, PhD[2], Michael Jonathan Davies, Professor, PhD[1], Anne Sofie Siebuhr, PhD[2].

[1]Department of Biomedical Sciences, University of Copenhagen, Copenhagen, Denmark.

[2]Biomarkers and Research, Nordic Bioscience, Herlev, Denmark.

*Corresponding author: Pernille Juhl, Herlev hovedgade 207, 2730 Herlev, Denmark, Phone: +45 44 52 52 52, Fax: +45 4452 5251, pju@nordicbio.com.

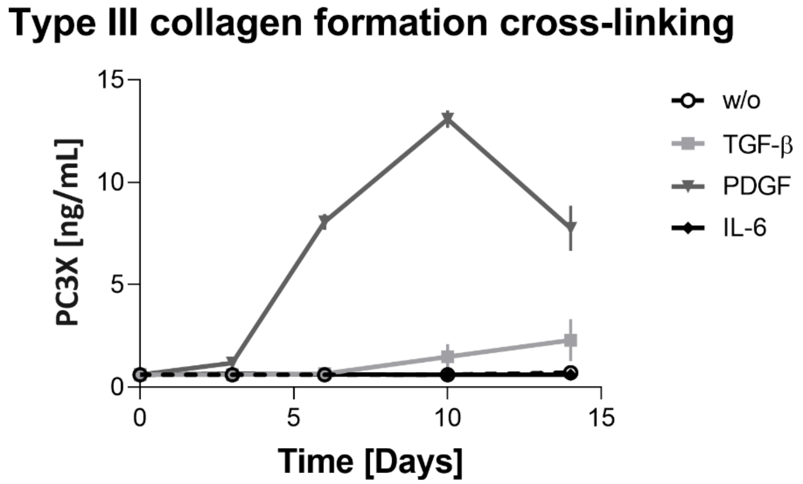


Figure S1. **Type III collagen is cross-linked over time.** Data are shown as mean ± SD.

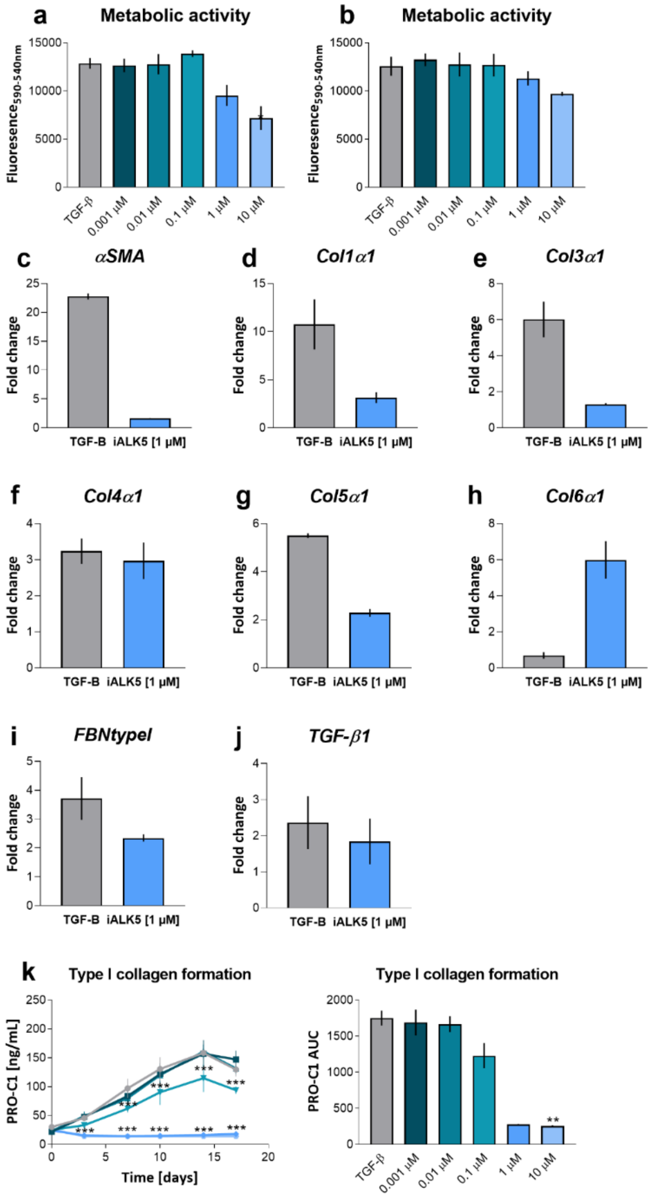


Figure S2. **TGF-β induced fibrosis can be modulated by iALK5.** (a) Metabolic activity of fibroblasts after addition of iALK5 from day 0. (b) Metabolic activity of fibroblasts after addition of iALK5 from day 7. (c) Gene expression of *αSMA*. (d) Gene expression of *Col1α1*. (e) Gene expression of *Col3α1*. (f) Gene expression of *Col4α1*. (g) Gene expression of *Col5α1*. (h) Gene expression of *Col6α1*. (i) Gene expression of *FBNtypeI*. (j) Gene expression of *TGF-β1*. (k) Type I collagen formation in response to TGF-β and iALK5 treatment form day 0. Four technical replicates were used to assess the metabolic activity and type I collagen formation, and two technical replicates were used to assess gene expression. Data are shown as mean ± SD. Data were analyzed by Kruskal-Wallis test and two-way ANOVA with Dunnett's multiple comparisons test. *: $P<0.05$, **: $P<0.01$, ***: $P<0.001$

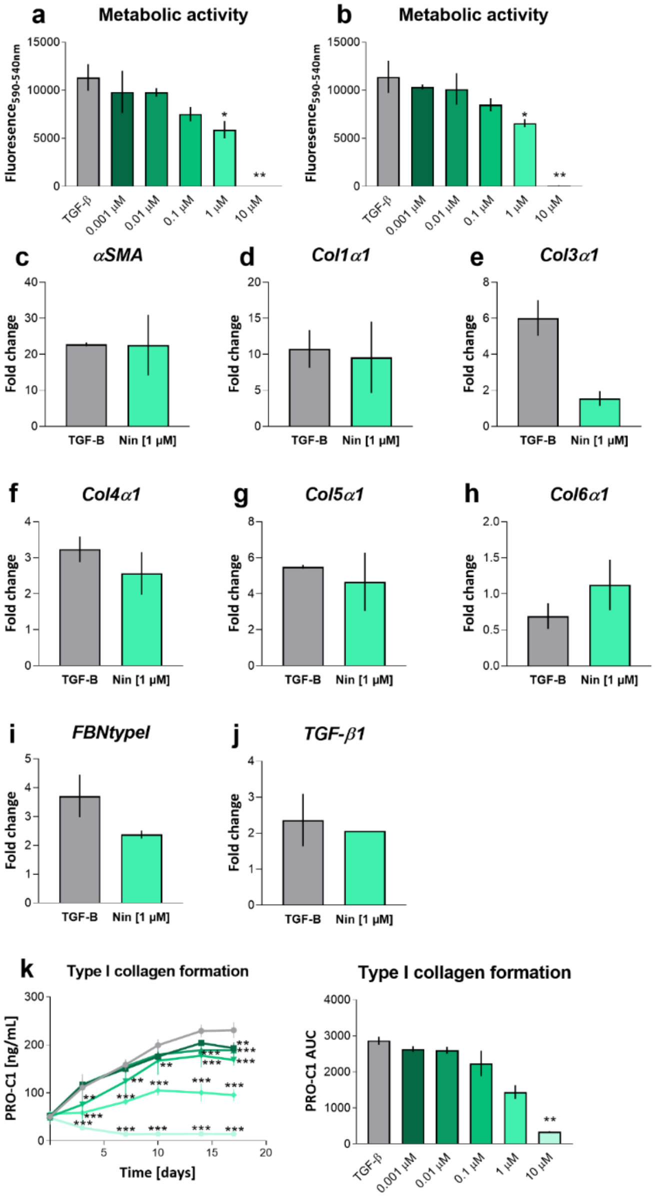


Figure S3. **TGF-β induced fibrosis can be modulated by nintedanib.** (a) Metabolic activity of fibroblasts after addition of nintedanib from day 0. (b) Metabolic activity of fibroblasts after addition of nintedanib from day 7. (c) Gene expression of *αSMA*. (d) Gene expression of *Col1α1*. (e) Gene expression of *Col3α1*. (f) Gene expression of *Col4α1*. (g) Gene expression of *Col5α1*. (h) Gene expression of *Col6α1*. (i) Gene expression of *FBNtypeI*. (j) Gene expression of *TGF-β1*. (k) Type I collagen formation in response to TGF-β and nintedanib treatment form day 0. Four technical replicates were used to assess the metabolic activity and type I collagen formation, and two technical replicates were used to assess gene expression. Data are shown as mean ± SD. Data were analyzed by Kruskal-Wallis test and two-way ANOVA with Dunnett's multiple comparisons test. *: P<0.05, **: P<0.01, ***: P<0.001.

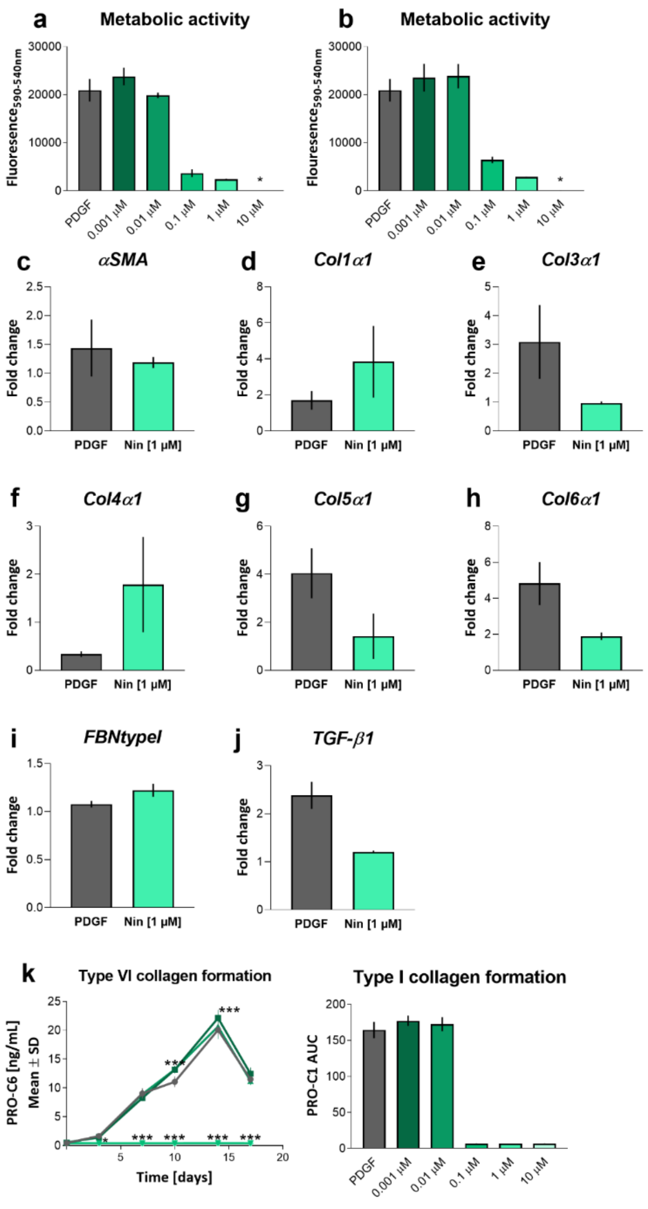


Figure S4. **PDGF induced fibrosis can be modulated by nintedanib.** (a) Metabolic activity of fibroblasts after addition of nintedanib from day 0. (b) Metabolic activity of fibroblasts after addition of nintedanib from day 7. (c) Gene expression of *αSMA*. (d) Gene expression of *Col1α1*. (e) Gene expression of *Col3α1*. (f) Gene expression of *Col4α1*. (g) Gene expression of *Col5α1*. (h) Gene expression of *Col6α1*. (i) Gene expression of *FBNtypeI*. (j) Gene expression of *TGF-β1*. (k) Type VI collagen formation in response to PDGF and nintedanib treatment form day 0. Four technical replicates were used to assess the metabolic activity and type VI collagen formation, and two technical replicates were used to assess gene expression. Data are shown as mean ± SD. Data were analyzed by Kruskal-Wallis test and two-way ANOVA with Dunnett's multiple comparisons test. *: P<0.05, **: P<0.01, ***: P<0.001.
